# The Immunohistochemical Loss of H3K27me3 in Intracranial Meningiomas Predicts Shorter Progression-Free Survival after Stereotactic Radiosurgery

**DOI:** 10.3390/cancers14071718

**Published:** 2022-03-28

**Authors:** Serena Ammendola, Paola Chiara Rizzo, Michele Longhi, Emanuele Zivelonghi, Serena Pedron, Giampietro Pinna, Francesco Sala, Antonio Nicolato, Aldo Scarpa, Valeria Barresi

**Affiliations:** 1 Department of Diagnostics and Public Health, Section of Anatomic Pathology, University of Verona, 37134 Verona, Italy; serena.ammendola@univr.it (S.A.); paolachiara.rizzo@univr.it (P.C.R.); serena.pedron@univr.it (S.P.); aldo.scarpa@univr.it (A.S.); 2 Unit of Stereotactic Neurosurgery, Department of Neurosciences, Hospital Trust of Verona, 37134 Verona, Italy; michele.longhi@aovr.veneto.it (M.L.); emanuele.zivelonghi@aovr.veneto.it (E.Z.); antonio.nicolato@aovr.veneto.it (A.N.); 3 Unit of Neurosurgery, Department of Neurosciences, Hospital Trust of Verona, 37134 Verona, Italy; giampietro.pinna@aovr.veneto.it; 4 Department of Neurosciences, Biomedicines and Movement Sciences, Institute of Neurosurgery, University of Verona, 37134 Verona, Italy; francesco.sala@univr.it; 5 ARC-NET Research Centre, University and Hospital Trust of Verona, 37134 Verona, Italy

**Keywords:** meningioma, H3K27me3, stereotactic radiosurgery, progression, radiosensitivity, Gamma knife

## Abstract

**Simple Summary:**

In this study, we aimed to investigate whether the immunohistochemical expression of H3K27me3 in meningiomas might predict tumor progression after stereotactic radiosurgery (SRS) performed for residual or recurrent disease. In 39 intracranial meningiomas, H3K27me3 loss was significantly associated with tumor progression (*p* = 0.0143) and shorter PFS after SRS (*p* = 0.0036). These findings suggest that the loss of H3K27me3 in meningiomas may correlate to a weaker response to SRS.

**Abstract:**

The immunohistochemical loss of histone H3 trimethylated in lysine 27 (H3K27me3) was recently shown to predict recurrence of meningiomas after surgery. However, its association with tumor progression after stereotactic radiosurgery (SRS) is unexplored. To investigate whether H3K27 methylation status may predict progression-free survival (PFS) after SRS, we assessed H3K27me3 immunoexpression in thirty-nine treatment naïve, intracranial, meningiomas, treated with surgery and subsequent SRS for residual (twenty-three cases) or recurrent (sixteen cases) disease. H3K27me3 immunostaining was lost in seven meningiomas, retained in twenty-seven and inconclusive in five. Six of the seven meningiomas (86%) with H3K27me3 loss had tumor progression after SRS, compared to nine of twenty-seven (33%) with H3K27me3 retention (*p* = 0.0143). In addition, patients harboring a meningioma with H3K27me3 loss had significantly shorter PFS after SRS (range: 10–81 months; median: 34 months), compared to patients featuring a meningioma with retained H3K27me3 (range: 9–143 months; median: 62 months) (*p* = 0.0036). Nonetheless, tumor sagittal location was the only significant prognostic variable at multivariate analysis for PFS after SRS (*p* = 0.0142). These findings suggest a previously unreported role of H3K27me3 as a predictor of meningioma progression after SRS for recurrent or residual disease. Modulation of H3K27 methylation status may represent a novel therapeutic strategy to induce radiosensitization of meningiomas.

## 1. Introduction

Meningiomas represent the most frequent primary intracranial tumors, accounting for approximately 38% of all brain neoplasias [1].

The World Health Organization (WHO) histological grade [2] and the extent of surgical resection are prognostic for the recurrence risk of these tumors and are major elements of the adjuvant therapy decision-tree in patients with meningiomas [3]. In detail, adjuvant fractionated radiotherapy is recommended in WHO grade 3 and in partially resected WHO grade 1 and 2 tumors, while its benefit is controversial in patients with totally resected WHO grade 2 meningiomas [3]. Stereotactic radiosurgery (SRS) is used as an alternative adjuvant therapy for residual or recurrent meningiomas, although the best timing for its administration remains undefined [4,5].

A recent multicentric study on 271 patients with atypical (grade two) or malignant (grade 3) meningiomas showed that the tumor WHO grade or proliferation index might influence the response to SRS [5]. Indeed, patients harboring a meningioma with grade 2 histology or Ki-67 labeling index (LI) ≤ 15% had significantly longer progression-free survival (PFS) after SRS than those with a meningioma having grade 3 histology or Ki-67 LI > 15% [5]. This suggests that the histopathological features of meningiomas may not only be prognostically informative, but also predictive of therapy response. However, the PFS after SRS is widely variable even among patients with meningiomas having the same histological grade [6,7]. Therefore, additional factors that may predict the response to SRS could be relevant to identifying patients who would benefit from this treatment.

There is evidence that DNA methylation profiling may stratify meningiomas for recurrence risk [8]. Among DNA epigenetic modifications, the immunohistochemical loss of histone H3 trimethylated in lysine 27 (H3K27me3) was shown to be prognostically relevant in meningiomas [9,10,11], and its use was proposed to identify those patients who may benefit from adjuvant radiation or a more stringent clinical and radiological follow-up [10]. In a recent analysis on four secondary (recurrent) grade 3 meningiomas treated with SRS, we found that the lack of H3K27me3 immunohistochemical expression in the corresponding primary tumor was associated with a worse clinical outcome [12]. This suggests that the assessment of H3K27me3 immunoexpression may be useful to predict PFS after SRS treatment.

This study aims at analyzing the prognostic role of H3K27me3 immunoexpression in meningiomas treated with surgery and subsequent SRS for residual or recurrent disease.

## 2. Materials and Methods

### 2.1. Cases

A total of 39 patients (13 females and 26 males; age range: 35–86 years; mean age: 61.5 ± 10.4 years; median age: 64 years), who underwent surgery for intracranial meningioma and subsequent Gamma knife SRS for residual or recurrent disease, were retrospectively included in this study. The cohort included two cases previously analyzed in another study [12].

The inclusion criteria were: (i) SRS performed for a recurrent or residual, nonsyndromic, nonradiation-induced, intracranial meningioma; (ii) a minimum follow-up of 24 months after SRS treatment in case of no disease progression; (iii) availability of paraffin blocks.

The histological slides of all meningiomas were revised to assess WHO grade according to the 5th update of the central nervous system tumors classification [2].

Clinical features and data on the extent of surgical resection, SRS treatment, and tumor progression after SRS were retrieved using operatory registries and clinical records.

Patients were followed up with magnetic resonance imaging every three months. Tumor volume and maximal diameter were calculated at the time of SRS and at every follow-up visit. Tumor progression was defined following the criteria of the response assessment in neuro-oncology (RANO) [13]. PFS after SRS was the length of survival since SRS treatment to the detection of tumor progression or the last follow-up.

### 2.2. Radiosurgery Technique

Patients were treated with SRS using the Gamma knife (Elekta, Stockholm, Sweden) radiosurgery device available at our institution.

Technical details of the GK procedure were previously extensively described [14,15]. Briefly, the Leksell stereotactic frame is positioned on the patient’s head under local anaesthesia, and neuroradiological localization is routinely performed using stereotactic MRI with specific algorithms and sequences: 1-mm- isovoxel volumetric, T1 fat saturated, and steady-state gadolinium-enhanced images.

SRS procedure was performed with the GK Perfexion model. Three-dimensional treatment planning was developed using Leksell Gamma Plan (versions 4.12, 5.34, 8.3, and 10.1.1; Elekta Instruments, Stockholm, Sweden). Dose selection was at the discretion of the treating neurosurgeon, radiation oncologist and radiation physicist team. SRS was characterized by PD and MD intensity delivered in compliance with the brain stem, optic nerve, the chiasm, and the pituitary peduncle. In all cases, the prescription isodose volume covered all recognizable tumor without adding any margin.

SRS was defined as: (i) adjuvant, when it was applied to treat the surgical bed or residual tumor within six months since surgery; (ii) salvage-residual, when it was used to treat a progressive residual tumor; (iii) salvage-recurrent, when it was carried out to treat a recurrent tumor after gross total resection.

### 2.3. Ethics

This study was approved by Comitato Etico per la Sperimentazione Clinica delle province di Verona e Rovigo (protocol n. 40400, 19 July 2019).

### 2.4. Immunohistochemistry

Four-µm thick sections were cut from a representative paraffin block of all meningiomas and immunostained using an antibody against H3K27me3 (clone C36B11, Cell Signaling Technology, Danvers, MA, USA; dilution 1:200), by means of an automated immunostainer (Leica Biosystems, Newcastle, UK). H3K27me3 immunohistochemical expression was rated as previously described [16]: (i) retained, when nuclear staining was seen in ≥5% neoplastic cells; (ii) lost, when staining was absent in >95% neoplastic cells and present in internal positive controls (endothelium, neurons); (iii) inconclusive, when staining was absent in both normal and neoplastic cells.

### 2.5. Statistical Analysis

The chi-squared test was used to analyze the statistical correlations between H3K27me3 immunoexpression and tumor progression or the clinical–pathological features (age and sex of the patients; timing of SRS treatment; localization or WHO grade of the tumor; type of treated tumor; localization of post-SRS recurrence). The same test was applied to investigate the statistical correlations between tumor progression and clinical–pathological features.

PFS was assessed by the Kaplan–Meier method, with the date of SRS treatment as the entry data and the length of survival to the detection of tumor progression as the end point. The Mantel–Cox log-rank test was applied to assess the strength of association between PFS and each of the parameters (age and sex of the patients; timing of SRS; location and H3K27me3 immunoexpression of the tumors) as a single variable. Successively, a multivariate analysis (Cox regression model) was used to determine the independent effect of each variable on PFS.

A probability (P) value less than 0.05 was considered significant. Statistical analyses were performed using MedCalc 12.1.4.0 statistical software (MedCalc Software, Mariakerke, Belgium).

## 3. Results

### 3.1. Cases and Radiosurgical Characteristics

**Figure 1:**
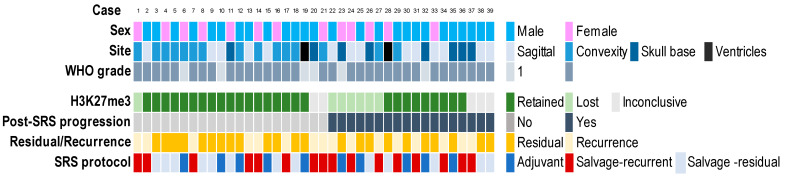
Clinical–pathological features and H3K27me3 immunoexpression of 39 intracranial meningiomas treated with surgery and subsequent stereotactic radiosurgery (SRS).

The clinical–pathological features of cases in the cohort are shown in Figure 1.

A total of 33 meningiomas (85%) were classified WHO grade 2 and 6 (15%) WHO grade 1. The anatomical location was the convexity in 15 cases (39%), the skull base in 8 (21%) and the cerebral ventricles in 2 (5%); 14 meningiomas were sagittal (35%).

Of the cohort, 16 (41%) patients had complete surgical resection of their meningioma and received salvage-recurrent SRS, and 23 (59%) patients had a tumor residue after surgery that was treated with adjuvant SRS in 10 cases (26%) (5 WHO grade 1 and 5 WHO grade 2), and with salvage-residual SRS in 13 (33%). In no cases, the postsurgical tumor cavity was treated with SRS in the absence of an identifiable tumor. No patients received radiotherapy prior to SRS.

The mean irradiated tumor volume was 6 cm^3^ (range 0.2–18 cm^3^). The prescription isodose line was 50% in all cases. The median margin dose was 13 Gy (IQ range: 13–14 Gy).

A total of 18 (46%) patients had disease progression after SRS (median PFS: 36 months; range: 9–81 months). These included 5/10 (50%) patients who received adjuvant SRS, 5/13 (38%) who were treated with salvage-residual SRS and 8/16 (50%) who underwent salvage-recurrent SRS. Tumor recurrence was in-field in 5 cases, at the edge of field in 3, and out-of-field in 10.

Twenty-one (54%) patients had no disease progression after SRS (median follow-up time: 62 months; range: 24–143 months). These included 5 patients who were treated with adjuvant SRS, 8 who received salvage-residual SRS and 8 who underwent salvage-recurrent SRS.

Meningioma progression after SRS treatment was significantly correlated with sagittal or skull base location (*p* = 0.011) (Table 1). The significant correlation between meningioma location and tumor progression was maintained in the subgroup of meningiomas with retained H3K27me3 (*p* = 0.0192).

### 3.2. Immunohistochemistry

H3K27me3 immunoexpression was inconclusive in five cases that had both normal and neoplastic cells unstained. The immunohistochemical assay was repeated using another paraffin block of each case, but it again yielded inconclusive results.

Seven (21%) meningiomas, including six WHO grade 2 and one WHO grade 1 tumors, lacking H3K27me3 staining in all the neoplastic cells, were classified as H3K27me3 negative.

**Figure 2:**
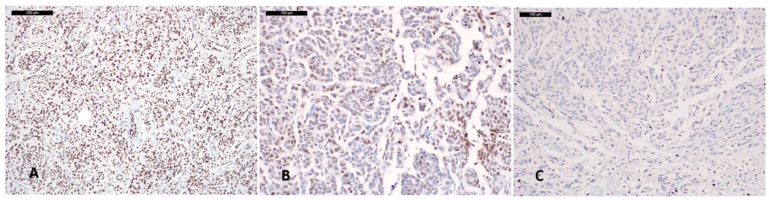
H3K27me3 Immunostaining in meningiomas: (**A**) meningioma classified H3K27me3 positive, with nuclear staining retained in all the neoplastic cells; (**B**) meningioma featuring retained H3K27me3 immunoexpression in 20% neoplastic cells; (**C**) meningioma classified H3K27me3 negative, showing unstained tumor cells and retained nuclear staining in the endothelial cells.

A total of 27 (79%) meningiomas, including 7 that retained H3K27me3 expression in all the neoplastic cells, 13 with >50% stained neoplastic cells and 7 with 25–50% stained tumor cells, were classified as H3K27me3 positive (Figure 1 and Figure 2).

Six of seven (86%) meningiomas (one WHO grade 1 and five WHO grade 2) with H3K27me3 loss had tumor progression after SRS, compared to 9 (one WHO grade 1 and eight WHO grade 2) (33%) with retained H3K27me3 (*p* = 0.0143).

H3K27me3 immunoexpression did not significantly correlate with any other clinical–pathological variables (Table 2).

### 3.3. Progression-Free Survival Analyses

**Figure 3:**
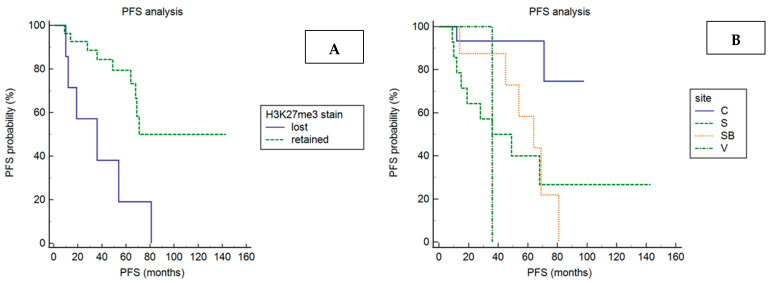
PFS analysis according to H3K27me3 immunoexpression (**A**) and tumor location (**B**). Kaplan–Meier curves show that PFS after SRS is significantly shorter in patients having a meningioma with H3K27me3 loss compared to patients harboring a meningioma with H3K27me3 retention (*p* = 0.0036) and in patients having a skull-based (SB) or sagittal (S) meningioma compared to patients harboring a meningioma located at the convexity (C) or intraventricular (V) (*p* = 0.023).

Patients harboring a meningioma with H3K27me3 loss (hazard ratio: 8.8; 95% confidence interval: 2–38.5; *p* = 0.0036), skull-based (hazard ratio: 6.3; 95% confidence interval: 1.9–21; *p* = 0.023) or sagittal (hazard ratio: 7.3; 95% confidence interval: 2.4–21.8; *p* = 0.023), had significantly shorter PFS than patients with a meningioma-retaining H3K27me3 stain or localized at the convexity (Figure 3; Table 3).

The statistical correlation between meningioma location and shorter PFS was maintained in the subgroup of cases with retained H3K27me3 (*p* = 0.0331). PFS length did not significantly correlate with any other clinical–pathological variables (Table 3).

In the multivariate analysis, including only covariates that were statistically significant in the univariates (H3K27me3 immunoexpression and tumor site), sagittal location (hazard ratio: 6.9; 95% confidence interval: 1.4–32.5; *p* = 0.0142), but not H3K27me3 immunoexpression, was an independent significant prognostic variable for PFS after SRS.

## 4. Discussion

In this study, we analyzed whether H3K27me3 immunohistochemical expression in intracranial meningiomas may predict progression risk and PFS after SRS treatment.

The prognostic role of H3K27me3 immunoexpression on the recurrence risk of surgically resected meningiomas was largely investigated in several studies, which showed that H3K27me3 loss is significantly more frequent in recurring meningiomas and significantly correlated with shorter recurrence-free survival in patients with WHO grade 1 and 2 tumors [9,10,11]. This has led us to consider the evaluation of H3K27me3 immunoexpression as a possible means to identify patients at increased recurrence risk after surgery and who could benefit from adjuvant treatments. Nonetheless, whether H3K27 methylation status may predict response to postsurgical SRS treatment was not assessed. Indeed, although a previous cohort included 59 patients treated with adjuvant radiotherapy, the correlation between H3K27me3 immunoexpression and PFS was not specifically investigated in this subgroup [9].

In this study on 33 WHO grade 2 and 6 grade 1 intracranial meningiomas treated with surgery and subsequent SRS, we found that H3K27me3 immunoexpression was lost in 7 cases, retained in 27, and, likely due to technical issues, inconclusive in 5 [11,17]. Considering only the cases with evaluable staining, H3K27me3 was lost in 20% WHO grade 1 and 21% WHO grade 2 meningiomas. Although these percentages are higher than those previously reported (1–4% in WHO grade 1 and 10.4–12% in WHO grade 2) [9,11], it should be noted that our cohort was enriched in recurring meningiomas, which more frequently lose H3K27me3 immunostaining.

H3K27me3 immunoexpression was not correlated with male sex, as opposed to previous observations [9], nor with any other clinical or pathological features, with the exception of tumor progression after SRS. Indeed, six of the seven cases (86%) with H3K27me3 loss progressed after SRS, compared to nine of twenty-seven (33%) with retained H3K27me3. In addition, the PFS after radiosurgery was significantly shorter in cases with H3K27me3 loss compared to those with retained H3K27me3.

These previously unreported findings suggest that the assessment of H3K27me3 staining could be useful to predict resistance to SRS in meningiomas. The negative prognostic role of H3K27me3 immunohistochemical loss was previously demonstrated in other tumors of the central nervous system such as posterior fossa ependymomas [18]. In detail, the immunohistochemical loss of H3K27me3 was associated with posterior fossa ependymomas of the methylation group B, which bear an unfavorable clinical outcome [18]. However, in contrast to what was found in patients with posterior fossa ependymomas, the H3K27me3 loss did not maintain a significant prognostic value at multivariate analysis in this cohort, and sagittal location was the only significant prognostic variable for PFS after SRS.

H3K27 trimethylation is mediated by the histone methyltransferase enhancer of zeste homolog 2 (EZH2) [19] and is removed by two demethylases KDM6A (UTX) and KDM6B (JMJD3) [20]. The correlation between H3K27 methylation status and radio- or chemosensitivity was previously analyzed in several other tumor types, with conflicting results. Loss of H3K27me3 was associated with radio- or chemoresistance in medulloblastomas [21] and colorectal carcinomas [22], while it was correlated with better response to chemotherapy in ovarian carcinoma and small-cell lung cancer [23,24]. This suggests that H3K27 methylation might play a different role in different cancers, likely due to the regulation of different signaling pathways and downstream target genes.

The mechanism by which low methylation levels of H3K27 could induce radio- or chemoresistance is unclear. We may hypothesize that chromatin condensation associated with histone methylation is an obstacle to the repair of DNA double strand breaks (DSBs) induced by ionizing radiation [25], while chromatin relaxation determined by histone demethylation facilitates DNA repair and contributes to cell survival after irradiation. In accordance, the inhibition of H3K27 demethylase sensitizes pontine glioma cell lines to radiotherapy and colorectal cancer patient-derived xenografts to chemotherapy [20,26]. Therefore, the inhibition of H3K27 demethylation could represent a strategy to enhance radiosensitivity in meningiomas.

On the one hand, the methylation status of H3K27 can influence chemo- or radiosensitivity, while on the other hand, chemo- or radiotherapy can modify the methylation status of H3K27. Indeed, irradiation produces H3K27me3 loss in tumor cell lines or patients’ xenografts via the activation of demethylases [25], and this could explain the reported loss of H3K27me3 in recurrent meningiomas after irradiation [12]. Since all meningiomas in the present study were treatment naïve, H3K27me3 loss cannot be a consequence of radiation exposure.

Aside from six cases with H3K27me3 loss, twelve additional meningiomas progressed after SRS, including three of four with inconclusive and nine of twenty-seven with retained H3K27me3 immunostaining. Since these cases were not re-operated after progression, we cannot determine whether irradiation produced a change in H3K27 methylation status and following, acquired radioresistance. Of note, sagittal or skull-based tumor location remained significantly associated with tumor progression and shorter PFS in the subgroup of meningiomas with retained H3K27me3, suggesting its negative prognostic relevance irrespective of H3K27 methylation status.

## 5. Conclusions

We showed for the first time that H3K27me3 loss predicts shorter PFS after adjuvant, salvage-residual or salvage-recurrent SRS in patients with intracranial meningiomas. However, this study has some limitations. First, in the absence of cases with irradiated surgical cavities after gross total resection, we could not define whether H3K27me3 could predict the response to adjuvant SRS in this setting. Second, since this study considered a small-sized and retrospective cohort, our findings should be validated in larger, prospective studies. The understanding of the mechanisms by which H3K27me3 loss could induce radioresistance in meningiomas warrants further investigation, as it could provide novel therapeutic strategies to radiosensitize these tumors. Similarly, the analysis of H3K27me3 immunoexpression in large cohorts of primary meningiomas and paired recurrences could be helpful to understand whether irradiation may cause a change in H3K27 methylation status and subsequent radioresistance.

## Figures and Tables

**Table 1:** Statistical correlation between post-SRS tumor progression and clinical–pathological features of 39 intracranial meningiomas.

Parameter	Post-SRS Tumor Progression		*p*
	Absent	Present	
*Age*			
<65 years	9	8	
≥65 years	12	10	0.8401
*Sex*			
Male	13	13	
Female	8	5	0.1928
*Site*			
Convexity	13	2	
Sagittal	5	9	
Skull base	2	6	
Intraventricular	1	1	0.023
*WHO grade*			
1	4	2	
2	17	16	0.5839
H3K27me3 immuno-expression *			
retained	18	9	
lost	1	6	0.0036
*SRS*			
Adjuvant	5	5	
Salvage-residual	8	5	
Salvage-recurrent	8	8	0.3752
*Treated tumor*			
Residual	13	10	
Recurrence	8	8	0.2828

SRS: stereotactic radiosurgery; * Statistical analysis was performed excluding five meningiomas with inconclusive H3K27me3 staining.

**Table 2:** Statistical correlation between H3K27me3 immunoexpression and clinical–pathological features of 34 intracranial meningiomas with evaluable H3K27me3 immunostaining.

Parameter	H3K27me3 Immuno-Expression		*p*
	Lost	Retained	
*Sex*			
Male	3	4	
Female	4	8	0.1811
*Age*			
<65 years	3	12	
≥65 years	4	15	0.9408
*Site*			
Sagittal	2	12	
Convexity	3	9	
Skull base	2	4	
Intraventricular	0	2	0.6599
*WHO grade*			
1	1	4	
2	6	23	0.9723
*Post-SRS tumor progression*			
Absent	1	18	
Present	6	9	0.0143
*SRS*			
Adjuvant	2	8	
Salvage-residual	1	10	
Salvage-recurrent	4	9	0.4241
*Treated tumor*			
Residual	3	18	
Recurrence	4	9	0.2551
*Post-SRS recurrence*			
Edge of field	1	2	
In-field	2	2	
Out-of-field	3	5	0.8856

SRS: stereotactic radiosurgery.

**Table 3:** Univariate analyses for progression-free survival after SRS in 39 patients with intracranial meningiomas.

Parameter	H.R. (95% C.I.)	*p*
*Sex*		
Male	1	
Female	0.5 (0.2–1.4)	0.1928
*Age*		
<65 years	1	
≥65 years	0.9 (0.3–2.3)	0.8401
*Site*		
Convexity	1	
Sagittal	7.3 (2.4–21.8)	
Skull base	6.3 (1.9–21)	
Intraventricular	8.4 (0.5–145.2)	0.023
*WHO grade*		
1	1	
2	1.4 (0.4–5.1)	0.839
*H3K27me3 immuno-expression*		
Retained	1	
Lost	8.9 (2–38.6)	0.036
*Treated tumor*		
Residual	0.6 (0.2–1.6)	
Recurrence	1	0.2828
*SRS*		
Adjuvant	1.8 (0.5–5.9)	
Salvage-residual	1	
Salvage-recurrent	2.1 (0.7–6.2)	0.3752

H.R.: hazard ratio. C.I.: confidence interval. SRS: stereotactic radiosurgery.

## Data Availability

The data presented in this study are available on request from the corresponding author. The data are not publicly available due to privacy issues.
